# Design of a real-world, prospective, longitudinal, observational study to compare vortioxetine with other standard of care antidepressant treatments in patients with major depressive disorder: a PatientsLikeMe survey

**DOI:** 10.1186/s12888-023-04922-6

**Published:** 2023-06-26

**Authors:** Subhara Raveendran, Deepshikha Singh, Mary C. Burke, Alicia H. McAuliffe-Fogarty, Sagar V. Parikh, Roger S. McIntyre, Anit Roy, Michael Martin, Lambros Chrones, Mark G. A. Opler, Chris Blair, Maggie McCue

**Affiliations:** 1 PatientsLikeMe, LLC, 6 Liberty Square, Suite 2602, Boston, MA 02109 USA; 2 Department of Psychiatry, Michigan Medicine, University of Michigan, 4250 Plymouth Road, Ann Arbor, MI 48109 USA; 3 Department of Psychiatry, University of Toronto and Centre for Addiction and Mental Health, 1451 Queen Street West, Toronto, ON M6R 1A1 Canada; 4 University Health Network, Mood Disorders Psychopharmacology Unit, 399 Bathurst St., Toronto, ON M5T 2S8 Canada; 5 Takeda Pharmaceuticals U.S.A., Inc., 95 Hayden Ave, Lexington, MA 02421 USA; 6 WCG Clinical Endpoint Solutions, 3 Park Avenue, New York, NY 10016 USA; 7 PANSS Institute, 19 Crotty Court, Monroe, NY 10950 USA

**Keywords:** PatientsLikeMe, Major depressive disorder, Patient-centric measures, Goal attainment, Vortioxetine, Shared decision-making

## Abstract

**Background:**

Major depressive disorder (MDD) is a recurrent psychiatric condition that presents challenges in responding to treatment and achieving long-term remission. To improve outcomes, a shared decision-making treatment approach with patient and healthcare practitioner (HCP) engagement is vital. PatientsLikeMe (PLM), a peer community of patients, provides information on MDD, symptoms, and treatment through forums and resources, helping patients stay engaged in their treatment journey. Data on PLM can be harnessed to gain insights into patient perspectives on MDD symptom management, medication switches, and treatment goals and measures.

**Methods:**

This ongoing, decentralized, longitudinal, observational, prospective study is being conducted using the PLM platform in two parts, enrolling up to 500 patients with MDD in the United States aged ≥ 18 years to compare vortioxetine with other monotherapy antidepressants. The first qualitative component consists of a webinar and discussion forum with PLM community members with MDD, followed by a pilot for functionality testing to improve the study flow and questions in the quantitative survey. The quantitative component follows on the PLM platform, utilizing patient-reported assessments, over a 24-week period. Three surveys will be conducted at baseline and weeks 12 and 24 to collect data on patient global impression of improvement, depression severity, cognitive function, quality of life (QoL) and well-being, medication satisfaction, emotional blunting, symptoms of anhedonia and resilience, as well as goal attainment. Quantitative results will be compared between groups. The qualitative component is complete; patient recruitment is underway for the quantitative component, with results expected in late 2023.

**Discussion:**

These results will help HCPs understand patient perspectives on the effectiveness of vortioxetine versus other monotherapy antidepressants in alleviating symptoms of MDD and improvements in QoL. Data from the PLM platform will support a patient goal-based treatment approach, as results can be shared by patients with their HCPs, providing them with insights on patient-centric goals, treatment management and adherence, as well as allowing them to observe changes in patient-related outcomes scores. Findings from the study will also help to optimize the PLM platform to build scalable solutions and connectivity within the community to better serve patients with MDD.

## Background

Depression is one of the largest causes of disability, accounting for a global disease burden of 4.3% [1]. Major depressive disorder (MDD) is the most prevalent and debilitating mental health disorder worldwide [2]. As of 2020, an estimated 21 million adults in the United States (US) had at least one major depressive episode, which represents 8.4% of all US adults [3]. The economic burden of MDD in the US is an estimated $326 billion, of which treatment cost is estimated to constitute only about 11% of that amount. The biggest contributors to this burden are comorbidities, mortality, and MDD-related workplace costs [4, 5]. The high rate of impairment caused by MDD, which includes poor health conditions, comorbidities, and mortality, is an issue of urgent concern for the US population [6]. Symptoms of MDD, such as the inability to maintain relationships and engage in leisure activities, are disabling for the patient, significantly impairing their daily life and functioning [7]. Emotional symptoms, such as depressed mood and anhedonia, are often accompanied by cognitive and physical dysfunction [8].

Despite clinical and therapeutic advances over the years, a large proportion of patients treated with antidepressants continue to face challenges in responding to treatment or achieving remission. Patients often require multiple treatments to find any benefit, and those with a greater number of different therapies can be more resistant to treatment [9]. These challenges often arise as a result of treatment approaches that seek acute symptomatic relief rather than the achievement of a long-term solution [9]. In addition, the definition of remission in MDD usually encompasses only traditional constructs such as sadness, anhedonia, and pessimistic thoughts. However, patients often perceive improvement in other outcomes, such as psychosocial and occupational functionality, as more meaningful [10–13].

Treatment nonadherence is another major challenge and may be due to varying education levels, side effects, poor interaction between the patient and healthcare provider (HCP), and culturally induced negative attitudes toward mental illness [14]. Furthermore, residual symptoms that persist despite antidepressant therapy can increase the risk of relapse and recurrence [15]. Recent literature has emphasized the need for an integrative and collaborative care approach to mitigate some of these issues and improve the treatment paradigm for patients with MDD [4].

### Measurement-based care (MBC) and shared decision-making (SDM)

The American Psychiatric Association provides clinical guidelines that recommend MBC for overcoming some of the challenges of treating depression. MBC can potentially enhance quality of care and improve clinical outcomes by including evaluations of quantitative symptom measures, level of functioning, and quality of life (QoL) [16]. MBC provides a systematic framework for evidence-based practice in monitoring routine outcomes and has shown significant benefits in treating a range of psychiatric disorders. Specifically, MBC allows clinicians to individualize treatment based on the patients’ symptoms and severity, identify non-responders, detect residual symptoms, and encourage treatment adherence through better patient engagement [17, 18].

Typically, MBC is characterized by routine administration of validated scales with scores that provide clinician- or patient-reported outcomes (PROs). These scores are used to inform SDM during treatment management [17], promoting clinician-patient engagement and encouraging patients to actively participate in their care, thus influencing treatment outcomes. Apart from better clinical outcomes, SDM positively affects patient satisfaction and adherence, measures increasingly considered to be part of treatment effectiveness [19].

One approach to SDM is for patients and clinicians to discuss individual treatment goals collaboratively. The Goal Attainment Scale adapted for Depression (GAS-D) is an instrument that provides a structured approach to setting and measuring progress toward the attainment of treatment goals. This allows for assessment of patient-centric goals that may not be captured with more traditional scales [10].

Patient engagement has been linked to better treatment outcomes, helping patients feel equipped, empowered, and enabled to deal with the challenges of MDD. In line with this, digital health platforms are low–resource-intensive, easily disseminated tools that help patients take ownership of their individual healthcare journeys [20]. Digital platforms have helped increase understanding of mental illness and may have a role in improving outcomes for patients with chronic mental health conditions. Real-time data obtained through these platforms can guide decision-making, course of treatment, and early intervention [21].

### PatientsLikeMe (PLM) platform

PLM is a web-based interactive digital health platform that is powered by patients and helps empower them to improve their lived experience. More than 850,000 members diagnosed with various conditions are members of PLM, including > 62,000 members with MDD, 50% of whom report MDD as a primary condition. The platform allows patients to share detailed computable information about their health, symptoms, treatments, and goals with other patients, and to learn from others’ experiences [22].

**Fig. 1:**
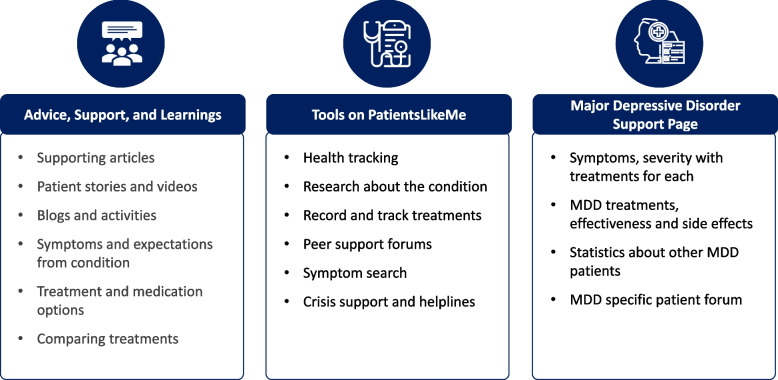
Key highlights of the PatientsLikeMe platform. Abbreviation: MDD = major depressive disorder

PLM is designed to help patients understand their conditions in the context of a peer-to-peer community. The PLM community gives patients with MDD a channel to receive education about their condition and what symptoms they can expect. It also provides information about available treatment options and offers a supportive arena to communicate with peers [22, 23]. The platform offers data-led intelligence to help patients better understand their health, identify treatment options, and nurture their shared journeys of healing. PLM enables patients to build resiliency and manage the complexity of their health, setting a new standard for holistic healing through personalized psychosocial interventions. PLM has helped patients make informed decisions, particularly about side-effect management, as observed through a cross-sectional online survey study, which demonstrated that patients were able to manage their symptoms better and experience greater benefit through PLM resources [19]. There is a vast amount of data available on PLM that can be harnessed to improve, design, and conduct trials in clinical and real-world settings [24]. These real-world research studies offer a way to understand PROs, quantify symptoms, and enable patient education and decision-making [22]. The key highlights of the PLM platform are presented in Fig. 1. PatientsLikeMe LLC receives research funding from various pharmaceutical and commercial partners and the site is free to use for members. However, PLM collaborates with pharmaceutical companies and medical device makers by offering research services using deidentified data.

### Patient perspective in MDD treatment

To fully understand the subjective experience of MDD, it is vital to assess cognitive function in patients [25]. Cognitive dysfunction is common in patients with MDD; however, the clinical setting offers no formal tools to routinely check for cognitive ability. Thus, studies that collectively look at clinical remission along with subjective symptom improvement become key to meeting this need. Additionally, HCPs cannot fully account for patient experience because of the burdensome depression assessment process and the lack of SDM. It is essential to understand the patient’s perspective regarding their symptoms and priorities to establish meaningful improvement through treatment [25]. Platforms such as PLM can serve as a bridge to connect researchers with real-world patient evidence and perspectives, thus emphasizing the need for MBC and SDM.

The goal of this study is to understand the outcomes of MDD in patients treated with monotherapy antidepressants such as vortioxetine, employing SDM and a goal-setting approach. Vortioxetine is a multimodal antidepressant that has demonstrated efficacy, safety, and tolerability in adults for the treatment of MDD [26]. In addition, patients receiving vortioxetine have shown improvement in cognitive symptoms, functionality, and overall QoL, along with beneficial effects in other physical symptoms such as sexual dysfunction, sleep disturbances, anxiety, pain, and weight loss [27]. Improvements in these residual symptoms are often associated with patients achieving full recovery [27].

### Rationale and aim of the study

In this ongoing, observational, real-world, prospective, decentralized study, we aim to gain insight and better understanding of the treatment experience of patients living with MDD, including symptom management, side effects, medication switches, and any treatment goals. Patients are provided with assessments within the PLM platform that support MBC, potential SDM, and tools to help them track their health-related goals, such as mood, cognitive symptoms, emotional well-being, and function. The primary objective of the study is to assess Patient Global Impression of Improvement (PGI-I) scores with vortioxetine at 12 weeks compared with other monotherapy antidepressant treatments following a new start or switch. Secondary objectives include measurement of changes between groups in MDD symptoms and severity, cognitive function, well-being, emotional blunting, resiliency, medication satisfaction, QoL, goal attainment, and patient engagement using the PLM platform. Additional areas of exploration will focus on aspects of the patient journey, such as medication adherence, reason for switching medication, compliance, and barriers to treatment.

## Methods

### Study design

**Fig. 2:**
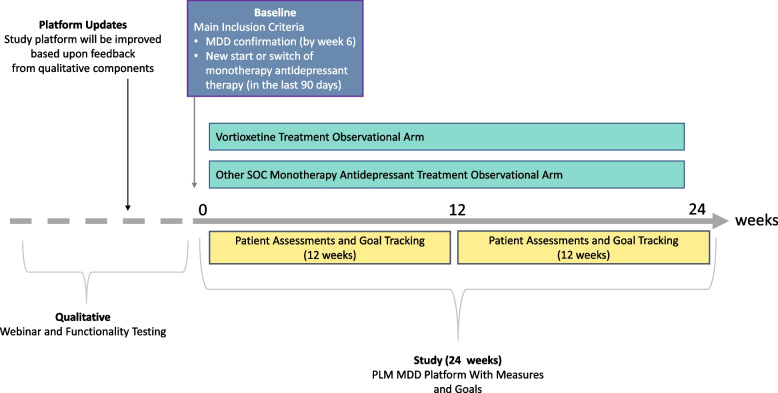
Study design. Abbreviations: MDD = major depressive disorder; PLM = PatientsLikeMe; SOC = standard of care

This ongoing, mixed-method, longitudinal cohort study, conducted in the PLM online patient community, analyzes overall improvement with vortioxetine at 12 weeks compared with other monotherapy antidepressants following a new start or switch of treatment using the PGI-I. This study consists of an initial qualitative educational webinar and a quantitative prospective analysis using patient-reported assessments along with goal setting and goal tracking over two 12-week periods (Fig. 2).

### Qualitative study

The initial qualitative study was planned as a developmental phase, including a webinar and survey using the PLM platform, with a focus on usability, functionality, and value to the patient, which inform changes to the study flow and quantitative survey questions. The educational outreach webinar provided an overview of treatment and management of MDD in the PLM platform, followed by a discussion forum on MDD with 13 participants. The qualitative component included a functionality test with another 10 participants who were given an overview of the study and a walk-through of the study platform, including the quantitative survey and PROs. These patients were asked to provide feedback on components of the PLM platform related to study flow, usability of the platform, PLM engagement, and current health data tracking including new goal setting, goal-tracking features, and ways to improve the study flow and quantitative survey.

### Quantitative study

Three quantitative surveys at baseline, week 12, and week 24 will be conducted to collect the data. The quantitative survey uses the PLM platform to track important health information such as mood, well-being, cognitive impairment, function, QoL measures, and goal attainment. This platform also consists of patient-facing educational support and videos to set and track patient goals with their HCPs. A doctor visit guide is available for patients so they can relay important health information, as well as their goals and PROs, from the PLM platform to their care team, either electronically or face-to-face.

### Study population

This study will include up to 500 members of the PLM platform with MDD who are US residents aged ≥ 18 years. For the qualitative component of the study, 13 patients were enrolled into the webinar as well as in the question-and-answer (Q&A) portion of the qualitative analysis. An additional 10 patients participated in the testing of the survey instrument. At least 480 patients will be recruited for the quantitative survey so that a minimum of 378 patients can be evaluated at week 12 for the primary objective with targeted recruitment ratios of 2:1 for other standard of care (SOC) monotherapy to vortioxetine.

### Inclusion and exclusion criteria

This study consists of members of the PLM platform who have met the inclusion and exclusion criteria. Patients were included in the qualitative study group if they self-reported their MDD diagnosis on the PLM platform and are currently taking antidepressant monotherapy. For the quantitative study group, eligible patients must have a diagnosis of MDD by week 6, confirmed through an electronic medical record (EMR) in the PLM profile or physical medical records provided by the patient, or a clinical confirmation by the patient’s HCP. Patients without a confirmation of diagnosis will be excluded from the study. Additionally, patients in the quantitative group must have a baseline 9-item Patient Health Questionnaire (PHQ-9) score ≥ 5 and should have either recently started or changed antidepressant monotherapy within the last 90 days before consent. Patients with a reported diagnosis of bipolar disorder, schizophrenia, schizoaffective disorder, or post-traumatic stress disorder will be excluded from this study.

### Study procedures and measurements

#### Qualitative assessment

##### Webinar and Q&A forum

A patient-friendly webinar developed by PLM designed to engage patients and provide avenues for discussion was available on the platform. Topics included an overview of MDD, including symptoms, diagnosis, treatments, tips, management strategies, and an understanding of the PLM platform. It also included an introduction to the GAS-D approach and guidance for patients about when they can request their physician to switch antidepressants. Following the webinar, a Q&A forum was held to encourage additional questions and discussions with the goal of capturing concepts and themes that will improve the quantitative component. The Q&A forum was supported by senior medical advisors from PLM and made available for 7 days to view after the webinar. Patients were contacted through a PLM private message or email for feedback on improving the quantitative component.

##### Usability and functionality testing

Another 10 patients were invited to participate in testing the quantitative survey. The aim was to understand what people with MDD perceive as important reasons for participating in a research study and to learn about their expectations while uncovering pain points, frustrations, and barriers about the overall experience and how they would improve it. This served as a pilot to improve the study design and questionnaires and to contribute to the improvement of the platform itself. Insights gained helped inform PLM about the patient perspective on tools used in the study, including GAS-D, PROs, patient-HCP engagement, and specific questions related to the functionality of the PLM platform to identify strengths and limitations. Evaluation of how participants used the study tool to complete study-specific tasks as well as feedback for suggested changes marked completion of this part of the study. Patients’ expectations for the PLM platform and any suggested platform enhancements make up an important endpoint of the study.

#### Quantitative assessment

##### MDD confirmation

The presence of MDD will be confirmed by reviewing each patient’s diagnostic criteria and current treatment by accessing EMRs through their PLM profile. A directed keyword search will be performed on these records to assess the description and severity of the condition. If a patient does not consent to give access or their condition is not available on EMRs, they have the option to share medical records showing a diagnosis of MDD. Alternatively, they can download the form from the study platform that will allow their clinical practitioner to confirm their MDD diagnosis and antidepressant monotherapy treatment. The PLM team will then look for markers such as disease description to confirm the presence or absence of MDD.

##### Patient demographics and background

**Fig. 3:**
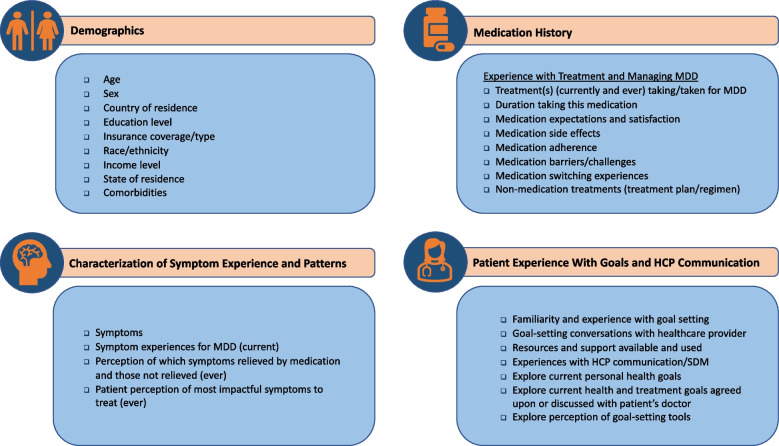
Patient demographics and insights captured during the study. Abbreviations: HCP = healthcare provider; MDD = major depressive disorder; SDM = shared decision-making

Information on patient demographics, symptom characteristics, medical history, and experience with treatment, goal attainment, and communication with their HCP will be collected (Fig. 3).

##### PLM web-based assessments

Patient measures: A set of surveys including 6 assessment scales will be posted on the PLM platform to capture patient-rated measures of depression, response to therapy using the PGI-I, cognitive functioning using the 5-item Perceived Deficits Questionnaire–Depression (PDQ-D5), QoL using the 5-item World Health Organization Well-being Index (WHO-5), depression severity using the PHQ-9, and life satisfaction using the Quality of Life Enjoyment and Satisfaction Questionnaire–Short Form (Q-LES-Q-SF) (Table 1).

**Table 1:** Patient assessments for primary and secondary outcomes

**Primary**	**Patient baseline reflection on current depression status (baselining for PGI-I)**
	**PLM Global Medication Satisfaction Questionnaire**
	• PGI-I Score
**Secondary**	**Subjective Cognitive Impairment**
	• PDQ-D5
	**QoL/Patient Well-being**
	• WHO-5
	**Life Satisfaction Questionnaire**
	• Q-LES-Q-SF
	**Depression Severity**
	• PHQ-9
	**Resilience** ^a^
	• CD-RISC-10
	**Emotional blunting (example questions on Likert scale: Never, Seldom, Not Sure, Sometimes, Always)** ^a^
	• My depression symptoms seem to make me not care about things that I should
	• My current antidepressant treatment seems to make me feel emotionally cut off from my family and friends
	• My current antidepressant treatment dulls my emotions and keeps me from having positive or negative feelings
	**Symptoms of anhedonia** ^**a**^
	• I would enjoy being with family or close friends
	• I would find pleasure in my hobbies and pastimes

^a^Not required, but patients are encouraged to complete

*CD-RISC-10* 10-Item Connor-Davidson Resilience Scale, *PDQ-D5* 5-Item Perceived Deficits Questionnaire–Depression, *PGI-I* Patient Global Impression of Improvement, *PHQ-9* 9-item Patient Health Questionnaire, *PLM* PatientsLikeMe, *Q-LES-Q-SF* Quality of Life Enjoyment and Satisfaction Questionnaire–Short Form, *QoL* quality of life, *WHO-5* 5-Item World Health Organization Well-being Index

In addition, sections with questions related to background, emotional blunting, anhedonia, and resiliency will also be available; however, completion of this section and goal tracking are not required for patients but rather encouraged.

Platform goal tracking: After baseline assessments, patients will be asked to create up to 3 specific, measurable, achievable, realistic, and time-bound (SMART) goals on the PLM platform using the GAS-D approach. Educational videos on how to set and track goals will be available throughout the study period. Patients will track the progress of their goals every week and will be able to see their aggregated goal progress. After 12 weeks, patients will be asked to provide final progress updates on the 3 goals they set for goal attainment calculations. Then they will be asked to set 3 new goals or to update previous goals to track from week 12 to week 24.

#### Data collection, management, and statistical analyses

Patient demographic data will be collected at baseline, and the web-based assessment data and some background data will be collected at weeks 12 and 24. The schedule for these evaluations is presented in Table 2. Final analytic datasets, with no individual identifying information, will be shared with the study collaborator for future analytic use. Any publication or presentations of the results from the research will include only aggregated, deidentified information.

**Table 2:** Schedule of evaluations

**PLM-MDD** **Schedule of Evaluations**	**Screening and Webinar**	**MDD Forum**	**User Experience**	**Screening**	**Baseline**	Weeks 1–5	Week 6	Weeks 7–11	Week 12	Weeks 13–17	Week 18	Weeks 19–23	Week 24
						+6 days	+6 days	+6 days	+6 days	+6 days	+6 days	+6 days	+10 days
**Qualitative Component**													
Screening and webinar invites	**X**												
Webinar	**X**												
Screening and invites	**X**												
Qualitative consent		**X**	**X**										
Q&A forum		**X**											
Useability and functionality insights			**X**										
**Quantitative Component**													
**Background and Insights**													
Screening and invite emails				**X**									
Consent					**X**								
MDD diagnosis confirmation					**X**^**a**^								
Demographics (age, sex, race, ethnicity, weight, height, employment status, insurance class)					**X**								
Comorbidities					**X**								
Antidepressant treatment experience					**X**								
Characterization of symptom experience importance and patterns					**X**				**X**				**X**
Patient experiences: goals and HCP communication					**X**				**X**				**X**
Current antidepressant medication					**X**				**X**				**X**
**PLM Web-Based Assessments**													
PHQ-9				**X**			**X**		**X**		**X**		**X**
Baseline reflection on current depression status					**X**								
PGI-I									**X**				**X**
Q-LES-Q-SF					**X**				**X**				**X**
PDQ-D5					**X**				**X**				**X**
WHO-5					**X**				**X**				**X**
Emotional blunting questions					**X**				**X**				**X**
SHAPS questions					**X**				**X**				**X**
CD-RISC-10					**X**				**X**				**X**
Goal setting/tracking questions					**X**				**X**				**X**
Goal setting					**X**				**X**				
Goal attainment tracking						**X**	**X**	**X**	**X**	**X**	**X**	**X**	**X**
Goal attainment scale for depression									**X**				**X**

^a^6 weeks for MDD confirmation

*CD-RISC-10* 10-Item Conner-Davidson Resilience Scale, *HCP* healthcare provider, *MDD* major depressive disorder, *PDQ-D5* 5-item Perceived Deficits Questionnaire–Depression, *PGI-I* Patient Global Impression of Improvement, *PHQ-9* 9-item Patient Health Questionnaire, *PLM* PatientsLikeMe, *Q&A* question-and-answer, *Q-LES-Q-SF* Quality of Life Enjoyment and Satisfaction Questionnaire–Short Form, *SHAPS* Snaith-Hamilton Pleasure Scale, *WHO-5* 5-item World Health Organization Well-being Index

For the qualitative analysis, themes arising from any free-text data were qualitatively evaluated and presented as frequencies in tables using a directed content analysis approach. A coding system for thematic analysis was created by PLM team members. The quantitative analysis will be summarized using descriptive statistics for patient characteristics by age, sex, condition-related characteristics, and treatments. For continuous variables, descriptive statistics will include the number of observations (N), mean and SD (mean, SD), median and interquartile range (median, [Q1, Q3]), and minimum and maximum values (min, max). For categorical variables, descriptive statistics will include the frequency and relative percentage of values in each category. Upon review of data, chi-square statistics will be used for categorical variables, while two sample *t*-tests and the analysis of variance procedure will be used to assess continuous variables between/among groups. To address confounding, various methods used in observational studies, such as stratification, corresponding to the levels of potential confounders (e.g., age groups and sex) will be employed as applicable. A multivariable regression analysis also will be used to control confounding. A total of 480 patients are planned to be recruited, to have at least 378 patients complete the week 12 survey after accounting for 15% of responders at week 12 (PGI-I < 2) and 20% attrition.

## Results

The webinar, Q&A forum, and qualitative component of the study have been completed, and completion of the quantitative component is scheduled for September 2023. The webinar received > 2000 clicks and > 200 views. The 10 patients enrolled for the usability testing were women aged 51 to 80 years. Results will be available in subsequent publications and are expected to provide insights into treatment experiences such as improvement in symptoms, cognitive ability, treatment satisfaction, emotional blunting, anhedonia, and overall well-being with vortioxetine versus other SOC monotherapy from a patient perspective in a real-world setting in patients with MDD. The goal achievement approach will shed light on how patients perceive their treatment goals and will help identify the improvements that are most meaningful to them. Another focus of this study will be to gain feedback from patients on how they perceive various functionalities on the PLM platform, such as sharing health data with HCPs and how this can be further improved to benefit patients. Thus, features such as goal setting and tracking may evolve to be permanently included as a function on the PLM platform, depending on patient feedback. Some study features may advance to become permanent PLM platform components based on results of the interest and engagement patients show in setting and tracking their goals and the feedback provided during functionality testing.

## Discussion

We believe that the methodology of this study can add value to the MDD community because of its unique design that uses a combination of patient education, surveys, and a set of quantitative assessments captured with PROs. The outcomes of this study will be patient-driven, and as it is hosted on the PLM platform, the data can offer real-world evidence, representing the population in a clinical setting. In addition, by using the goal-setting and goal-tracking approach in this study, we can better understand what patients with MDD consider to be their goals in recovery. Thus, key insights can be gained into patient perspectives of improvement in their daily functioning and well-being.

PLM is an online patient community that serves as a repository for patient-produced data, leveraging the power of social networking among patients with chronic, life-changing conditions [28]. The platform provides patients with resources to explore their condition, share experiences with peers, track their symptoms, identify treatment options, and share outcomes, thereby improving overall quality of care [23]. Digital platforms such as PLM provide patients with MDD with a greater understanding of the disease and can potentially improve outcomes in this population as a result of informed treatment decisions and improved medication adherence [21]. For patients with MDD, deidentified, computable data obtained through the PLM platform can be an effective measure of PROs, patient perspectives, and preferences [28]. Research studies conducted on PLM have the potential to be patient focused, as they capture patient-centric information and are easy to participate in because they are virtual.

The PLM platform enables the conduct of observational studies, which play a vital role in investigating treatment outcomes. In the primary care setting, observational studies help to inform SDM and patient engagement in their treatment [29]. The prospective design of this study will provide insights into the effectiveness of vortioxetine compared with other SOC treatments, as well as the use of digital technology for patient support in a real-world setting [30].

To fully understand goals and meaningful outcomes for patients with MDD, it is vital to identify the challenges that they face in the course of their treatment. Patients with MDD seeking treatment often experience improvement in symptoms related to mood much later than improvements in physical and cognitive symptoms. Some of the main reasons for treatment discontinuation cited by patients with MDD are lack of remission, low tolerability, and minimal engagement with their HCPs [31]. A majority of patients with MDD report residual functional impairment, which does not lead to long-term recovery [15, 32]. In the real-world RELIEVE study, vortioxetine was found to be safe and effective in improving functioning and treatment adherence in clinical practice. Patients also experienced clinically relevant improvements in depressive symptoms, cognitive symptoms and performance, and health-related QoL over the 6-month treatment period [33].

Further expanding on existing evidence, this study aims to provide data supporting patients’ perspectives of MDD symptom improvement and will assess patient-centric outcomes for mood, cognitive symptoms, emotional well-being, medication satisfaction, QoL, and goal engagement in new start-and-switch patient populations with long-term use of vortioxetine versus other SOC monotherapy. For patients with MDD, PROs can accurately represent clinical status, especially for the maintenance of long-term disease [34]. The PRO assessments used in this PLM study for symptom improvement (PGI-I), subjective cognitive impairment (PDQ-D5), treatment satisfaction (Q-LES-Q-SF), and depression severity (PHQ-9) can provide substantiating evidence for the use of these scales. The outcomes of this PLM study will help to elucidate the importance of patient engagement with HCPs and may provide a better way to develop tools for patients to be able to track, share, and be in control of their condition and treatment. By incorporating a patient-reported, goal-based approach, we aim to understand how receptive patients with MDD are to setting and sharing their goals with HCPs [35]. The evidence for the benefit of SDM and MBC for meaningful treatment outcomes in patients with MDD will be the cornerstone of this study.

### Strengths

Patients on the PLM platform represent a real-world population with comorbidities and standard treatment doses who are following a treatment adherence pattern representative of clinical settings. The unique advantage of enrolling patients from the general population, such as those from PLM, is that they are not bound by the stringent exclusion criteria of randomized controlled trials. The PLM platform has a large number of members with MDD as a primary condition, and this provides an opportunity to improve study enrollment [36]. Patients with MDD may have experienced various treatments and are in a better position to assess their improvements, leading to a better understanding of meaningful outcomes in the clinical setting. Although this is an observational study, adherence to treatment in the clinical setting is an important aspect of treatment from which to get accurate data.

### Limitations

Like all real-world evidence studies, this study involves self-reported data from patients and their encounters, thus limiting the generalizability and interpretation of results. There also could be a risk of missing variables of interest from records. As with other observational studies, this study can be confounded by patients’ selection and indication, as patients who choose to engage in a digital platform like PLM may be different from the general population. To mitigate some of the bias, confirmation of MDD diagnosis and treatment will be performed for patients reporting outcomes in the quantitative part of the study.

## Conclusion

These results will help HCPs understand patient perspectives on the effectiveness of vortioxetine compared with other monotherapy antidepressants in alleviating symptoms of MDD and improvements in QoL. Data from the PLM platform are planned to support a goal-based treatment approach to achieve SDM between patients and HCPs. HCPs can gain insights into patient-centric goals, treatment management, and adherence as well as observe the change in patient-related outcome scores, as patients have the option to download their results and share them with their HCPs. Findings from this study can help optimize the PLM platform to build scalable solutions and connectivity within the PLM community to better serve patients with MDD.

## Data Availability

Data sharing is not applicable to this article as it is a protocol, and no datasets were generated or analyzed. The statistical analysis plan can be made available upon request.

## Abbreviations

CD-RISC-10: 10-item Connor-Davidson Resilience Scale
EMR: Electronic medical record
GAS-D: Goal Attainment Scale adapted for Depression
HCP: Healthcare practitioner
MBC: Measurement-based care
MDD: Major depressive disorder
PDQ-D5: 5-item Perceived Deficits Questionnaire–Depression
PGI-I: Patient Global Impression of Improvement
PHQ-9: 9-item Patient Health Questionnaire
PLM: PatientsLikeMe
PROs: Patient-reported outcomes
Q&A: Question-and-answer
Q-LES-Q-SF: Quality of Life Enjoyment and Satisfaction Questionnaire–Short Form
QoL: Quality of life
SDM: Shared decision-making
SHAPS: Snaith-Hamilton Pleasure Scale
SMART: Specific, Measurable, Achievable, Realistic, and Time-bound
SOC: Standard of care
US: United States
WHO-5: 5-item World Health Organization Well-being Index

## References

[1] World Health Organization. Comprehensive mental health action plan 2013–2030. Geneva: 2021. https://www.who.int/publications/i/item/9789240031029. Accessed 23 May 2022.

[2] Gutierrez-Rojas L, Porras-Segovia A, Dunne H, Andrade-Gonzalez N, Cervilla JA (2020). Prevalence and correlates of major depressive disorder: a systematic review. Braz J Psychiatry.

[3] National Institute of Mental Health. Statistics: Major depression; 2022. https://www.nimh.nih.gov/health/statistics/major-depression. Accessed 22 Aug 2022.

[4] Greenberg PE, Fournier AA, Sisitsky T, Simes M, Berman R, Koenigsberg SH (2021). The economic burden of adults with major depressive disorder in the United States (2010 and 2018). Pharmacoeconomics.

[5] Lepine JP, Briley M (2011). The increasing burden of depression. Neuropsychiatr Dis Treat.

[6] Hasin DS, Goodwin RD, Stinson FS, Grant BF (2005). Epidemiology of major depressive disorder: results from the National Epidemiologic Survey on Alcoholism and Related Conditions. Arch Gen Psychiatry.

[7] IsHak WW, James DM, Mirocha J, Youssef H, Tobia G, Pi S (2016). Patient-reported functioning in major depressive disorder. Ther Adv Chronic Dis.

[8] Kennedy SH (2008). Core symptoms of major depressive disorder: relevance to diagnosis and treatment. Dialogues Clin Neurosci.

[9] Rush AJ, Trivedi MH, Wisniewski SR, Nierenberg AA, Stewart JW, Warden D (2006). Acute and longer-term outcomes in depressed outpatients requiring one or several treatment steps: a STAR*D report. Am J Psychiatry.

[10] McCue M, Parikh SV, Mucha L, Sarkey S, Cao C, Eramo A (2019). Adapting the goal attainment approach for major depressive disorder. Neurol Ther.

[11] McIntyre RS, Loft H, Christensen MC (2021). Efficacy of vortioxetine on anhedonia: results from a pooled analysis of short-term studies in patients with major depressive disorder. Neuropsychiatr Dis Treat.

[12] Trivedi MH, Rush AJ, Wisniewski SR, Nierenberg AA, Warden D, Ritz L (2006). Evaluation of outcomes with citalopram for depression using measurement-based care in STAR*D: implications for clinical practice. Am J Psychiatry.

[13] Trivedi MH, Daly EJ (2008). Treatment strategies to improve and sustain remission in major depressive disorder. Dialogues Clin Neurosci.

[14] Ho SC, Jacob SA, Tangiisuran B (2017). Barriers and facilitators of adherence to antidepressants among outpatients with major depressive disorder: a qualitative study. PLoS One.

[15] Halaris A, Sohl E, Whitham EA (2021). Treatment-resistant depression revisited: a glimmer of hope. J Pers Med.

[16] Gelenberg AJ (2010). A review of the current guidelines for depression treatment. J Clin Psychiatry.

[17] Zhu M, Hong RH, Yang T, Yang X, Wang X, Liu J (2021). The efficacy of measurement-based care for depressive disorders: systematic review and meta-analysis of randomized controlled trials. J Clin Psychiatry.

[18] Hong RH, Murphy JK, Michalak EE, Chakrabarty T, Wang Z, Parikh SV (2021). Implementing measurement-based care for depression: practical solutions for psychiatrists and primary care physicians. Neuropsychiatr Dis Treat.

[19] Loh A, Simon D, Wills CE, Kriston L, Niebling W, Harter M (2007). The effects of a shared decision-making intervention in primary care of depression: a cluster-randomized controlled trial. Patient Educ Couns.

[20] Birnbaum F, Lewis D, Rosen RK, Ranney ML (2015). Patient engagement and the design of digital health. Acad Emerg Med.

[21] Batra S, Baker RA, Wang T, Forma F, DiBiasi F, Peters-Strickland T (2017). Digital health technology for use in patients with serious mental illness: a systematic review of the literature. Med Devices (Auckl).

[22] Wicks P, Massagli M, Frost J, Brownstein C, Okun S, Vaughan T (2010). Sharing health data for better outcomes on PatientsLikeMe. J Med Internet Res.

[23] Borentain S, Nash AI, Dayal R, DiBernardo A (2020). Patient-reported outcomes in major depressive disorder with suicidal ideation: a real-world data analysis using PatientsLikeMe platform. BMC Psychiatry.

[24] US Food and Drug Administration. Real-world data (RWD) and real-world evidence (RWE) are playing an increasing role in health care decisions; 2022. https://www.fda.gov/science-research/science-and-research-special-topics/real-world-evidence. Accessed 26 Aug 2022.

[25] Chiauzzi E, Drahos J, Sarkey S, Curran C, Wang V, Tomori D (2019). Patient perspective of cognitive symptoms in major depressive disorder: retrospective database and prospective survey analyses. J Particip Med.

[26] Zhang X, Cai Y, Hu X, Lu CY, Nie X, Shi L (2022). Systematic review and meta-analysis of vortioxetine for the treatment of major depressive disorder in adults. Front Psychiatry.

[27] Christensen MC, Florea I, Lindsten A, Baldwin DS (2018). Efficacy of vortioxetine on the physical symptoms of major depressive disorder. J Psychopharmacol.

[28] Agency for Healthcare Research and Quality. PatientsLikeMe; 2017. https://www.ahrq.gov/workingforquality/priorities-in-action/patientslikeme.html. Accessed 12 Sept 2022.

[29] Jackson JL, Storch D, Jackson W, Becher D, O’Malley PG. Direct-observation cohort study of shared decision making in a primary care clinic. Med Decis Making. 2020;40(6):756–65. 10.1177/0272989X20936272 32639863

[30] Berger ML, Dreyer N, Anderson F, Towse A, Sedrakyan A, Normand SL (2012). Prospective observational studies to assess comparative effectiveness: the ISPOR good research practices task force report. Value Health.

[31] McNaughton EC, Curran C, Granskie J, Opler M, Sarkey S, Mucha L (2019). Patient attitudes toward and goals for MDD treatment: a survey study. Patient Prefer Adherence.

[32] Subramaniapillai M, Mansur RB, Zuckerman H, Park C, Lee Y, Iacobucci M (2019). Association between cognitive function and performance on effort based decision making in patients with major depressive disorder treated with vortioxetine. Compr Psychiatry.

[33] Mattingly GW, Ren H, Christensen MC, Katzman MA, Polosan M, Simonsen K (2022). Effectiveness of vortioxetine in patients with major depressive disorder in real-world clinical practice: results of the RELIEVE study. Front Psychiatry.

[34] Rush AJ, Trivedi MH, Carmody TJ, Ibrahim HM, Markowitz JC, Keitner GI (2005). Self-reported depressive symptom measures: sensitivity to detecting change in a randomized, controlled trial of chronically depressed, nonpsychotic outpatients. Neuropsychopharmacology.

[35] McCue M, Sarkey S, Eramo A, Francois C, Parikh SV (2022). Correction: Using the Goal Attainment Scale adapted for depression to better understand treatment outcomes in patients with major depressive disorder switching to vortioxetine: a phase 4, single-arm, open-label, multicenter study. BMC Psychiatry.

[36] Steinhubl SR, Wolff-Hughes DL, Nilsen W, Iturriaga E, Califf RM (2019). Digital clinical trials: creating a vision for the future. NPJ Digit Med.

